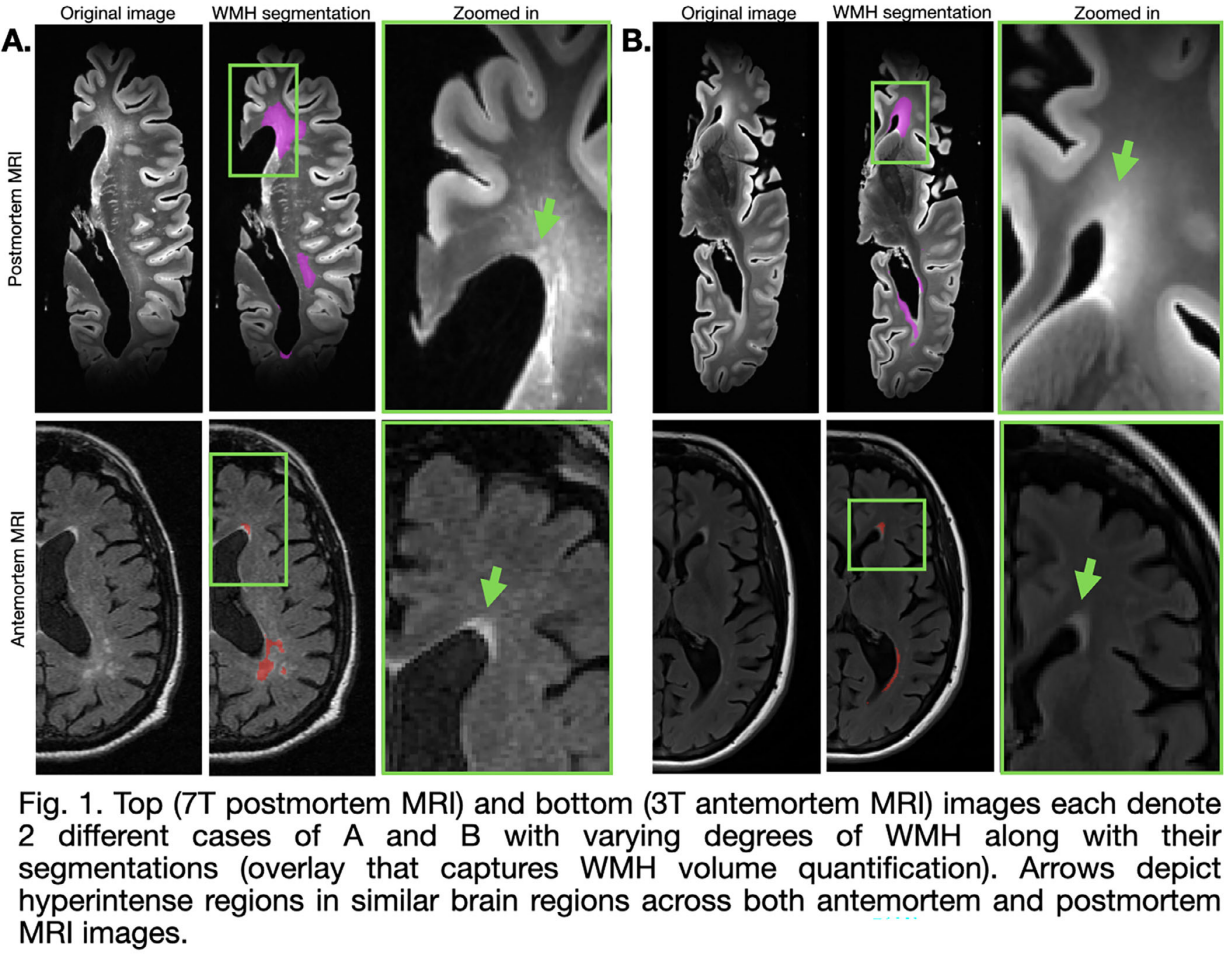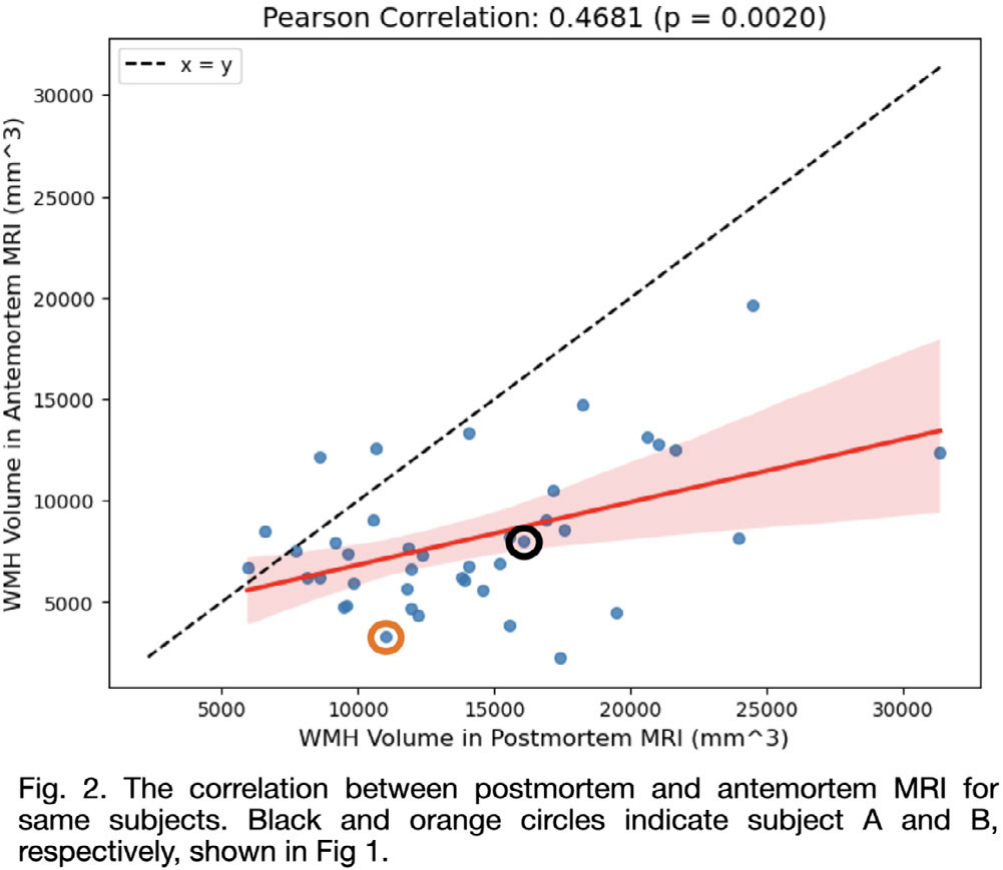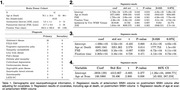# Assessing white matter hyperintensities on matched antemortem and postmortem MRI

**DOI:** 10.1002/alz70856_107162

**Published:** 2026-01-09

**Authors:** Zahra Khodakarami, Pulkit Khandelwal, Michael Tran Duong, Amanda E Denning, Sydney A. Lim, Eunice Chung, Alejandra Bahena, Karthik Prabhakaran, Gabor Mizsei, Theresa Schuck, Winifred Trotman, John L. Robinson, Daniel T Ohm, Adam Brickman, Eddie B. Lee, David J. Irwin, John A. Detre, M. Dylan Tisdall, Sandhitsu R. Das, David A. Wolk, Paul A. Yushkevich, Sheina Emrani

**Affiliations:** ^1^ Penn Image Computing and Science Laboratory (PICSL), University of Pennsylvania, Philadelphia, PA, USA; ^2^ University of Pennsylvania, Philadelphia, PA, USA; ^3^ Department of Pathology and Laboratory Medicine, Institute on Aging and Center for Neurodegenerative Disease Research, The Perelman School of Medicine at the University of Pennsylvania, Philadelphia, PA, USA; ^4^ Perelman School of Medicine at the University of Pennsylvania, Philadelphia, PA, USA; ^5^ Penn Frontotemporal Degeneration Center, Department of Neurology, Perelman School of Medicine, University of Pennsylvania, Philadelphia, PA, USA; ^6^ Taub Institute for Research on Alzheimer's Disease and the Aging Brain, New York, NY, USA; ^7^ Department of Pathology & Laboratory Medicine, Perelman School of Medicine, University of Pennsylvania, Philadelphia, PA, USA; ^8^ Penn FTD Center, University of Pennsylvania, Philadelphia, PA, USA; ^9^ Department of Neurology, Perelman School of Medicine, University of Pennsylvania, Philadelphia, PA, USA; ^10^ Institute on Aging, University of Pennsylvania, Philadelphia, PA, USA; ^11^ Center for Neurodegenerative Disease Research, Perelman School of Medicine, University of Pennsylvania, Philadelphia, PA, USA; ^12^ Penn Alzheimer's Disease Research Center, Perelman School of Medicine, Philadelphia, PA, USA

## Abstract

**Background:**

Postmortem MRI offers superior spatial resolution and contrast compared to antemortem MRI, enabling analysis of pathological processes underlying white matter hyperintensities (WMH). However, its correlation with antemortem WMH remains unclear due to postmortem changes, fixation effects, and tissue dehydration. This study compares WMH burden on antemortem and postmortem MRI in the same individuals to assess the suitability of postmortem MRI for structure‐pathology association studies.

**Method:**

7T T2‐weighted postmortem and 3T FLAIR antemortem MRIs were acquired from 41 individuals (Table 1). WMH segmentations were generated using Purple‐MRI for postmortem MRI and WMH‐SynthSeg for antemortem MRI (Figure 1). One hemisphere was scanned postmortem, and the corresponding antemortem hemisphere was used for WMH segmentation. Linear regression models examined (I) Antemortem WMH volume as a predictor of postmortem WMH, (II) age at scan as a predictor of antemortem WMH volume, and (III) age at death as a predictor of postmortem WMH volume. Nuisance covariates included antemortem/postmortem intervals (AMI/PMI) and fixation time, as appropriate.

**Result:**

Table 1 provides a breakdown of demographics and neuropathological diagnoses. A moderate, statistically significant correlation was found between antemortem and postmortem WMH volume (*r* = 0.47, *p* =  0.002;Figure 2), remaining significant after adjusting for age and nuisance covariates (Table 1). Both antemortem and postmortem WMH volumes were significantly associated with age (Table 4 [antemortem MRI] and 3 [postmortem MRI]), with a greater antemortem association in absolute terms (t=2.8 vs. t=2.58, n.s.). Other covariates were not significant. Postmortem WMH volume was, on average, 102±121% higher than antemortem, with only a weak association between their relative difference and AMI (r=0.27, *p* = 0.04), suggesting that WMH increase is due to greater lesion visibility in postmortem MRI rather than WMH growth. This aligns with histological findings showing WMH‐linked pathology in surrounding normal‐appearing white matter.

**Conclusion:**

This study highlights both a significant association between antemortem and postmortem WMH volume, and a significant bias towards larger WMH in postmortem MRI, above and beyond factors like AMI and fixation time. In subsequent work, we will use matched MRI and histology to study the contribution of vascular and neurodegenerative pathologies to the extent and appearance of WMH on antemortem and postmortem MRI.